# Mediation role of perceived social support and burnout on financial satisfaction and turnover intention in primary care providers: a cross-sectional study

**DOI:** 10.1186/s12913-021-06270-1

**Published:** 2021-03-19

**Authors:** Huosheng Yan, Lingzhi Sang, Hongzhang Liu, Cancan Li, Zijing Wang, Ren Chen, Hong Ding, Zhi Hu, Guimei Chen

**Affiliations:** School of Health Service Management, Anhui Medical University, No.81 Meishan Road, Shushan District, Hefei, 230032 China

**Keywords:** Perceived social support, Burnout, Financial satisfaction, Turnover intention, Structural equation modeling

## Abstract

**Background:**

Turnover intention is a major cause of reduced team morale and low work efficiency. It hinders work performance and reduces the quality of medical services. This study aimed to investigate the relationship between financial satisfaction and turnover intention and its mediators among primary care providers.

**Methods:**

Multi-stage random cluster sampling was used to select 1241 participants from four counties and three districts in Anhui province, China. Data were collected using a self-administered questionnaire. Turnover intention was assessed with a turnover intention assessment scale. Perceived social support and burnout were measured with the 12-item Perceived Social Support Scale and the Chinese version of the Maslach Burnout Inventory, respectively. Structural equation modeling was used for data analysis.

**Results:**

The findings showed high turnover intention among primary care providers (mean score 14.16 ± 4.337), and most providers reported low financial satisfaction (mean score 2.49 ± 0.990). The mean perceived social support score was 64.93 ± 13.229, and only 6.1% of primary care providers reported no burnout. Compared with participants with high financial satisfaction, those with low financial satisfaction were more likely to report higher turnover intention (β = − 0.216, *p* < 0.001), less perceived social support (β = 0.181, *p* < 0.001), and more severe burnout (β = − 0.123, *p* < 0.05). Turnover intention may be related to perceived social support (β = − 0.147, *p* < 0.001) and burnout (β = 0.239, *p* < 0.001). Furthermore, the effect of financial satisfaction on turnover intention was significantly mediated by perceived social support (β = − 0.027, *p* < 0.001) and burnout (β = − 0.029, *p* < 0.05).

**Conclusions:**

Turnover intention is associated with financial satisfaction, with this association mediated by perceived social support and burnout. A reasonable mechanism needs to be established to improve financial satisfaction and perceived social support and reduce burnout among primary care providers.

## Background

Strengthening the primary healthcare system is one of the five key targets of China’s healthcare reform, which started in 2009 [1]. This healthcare reform substantially improved access to and affordability of primary healthcare and reduced the disease burden [2]. However, primary healthcare providers face tremendous pressure caused by low levels of training and income. Although the Chinese government has invested many resources in healthcare reform, the reform of the drug system resulted in financial losses for primary care providers [3], however, incentives for primary care providers may be lacking [3]. Over the past decade, the number of primary care providers increased from 3.3 million to 3.8 million because of the continuing increase in demand for health services, but the proportion of primary care providers among all health workers declined from 40.0 to 32.6% [4]. The shortage of qualified primary care providers remains a critical challenge in healthcare and hinders the further development of the primary care system. Given the long time needed to train qualified health workers, strategies that can be implemented to reduce burnout and turnover intention among primary care providers are key concerns in the current situation.

Financial satisfaction refers to an individual’s subjective assessment of his financial status, which is more akin to psychological attributes than objective economic indicators [5]. Financial incentives act as motivation that affects practice behaviors of primary care providers and represents a policy option that can be introduced quickly to induce a behavioral response from primary care providers [6]. However, the same income level may be associated with differences in quality of life across different parts of China because of disparity in regional economic levels. Financial satisfaction is a subjective factor that can eliminate the differences associated with the level of regional economic development. Sufficient financial satisfaction can encourage primary care providers to provide better quality care to patients [7], and providers who provide the highest quality care may be those that are most likely to claim incentive payments [8]. Despite the increase in primary care providers’ income in recent years, the salary gap between primary care providers and doctors in general hospitals has widened over the past decade, and primary care providers earn around half as much as general hospital doctors [9]. Higher income satisfaction may reduce turnover intention among primary care providers [10] and reduce the pressure from human resources shortages. However, the primary healthcare system cannot attract younger primary care providers because of the low wages and minimal benefits [2]. Resolving the shortage of doctors working in primary care resulting from financial dissatisfaction has become a problem for policymakers.

Turnover intention is a major challenge in the primary healthcare system and is defined as the possibility of an employee resigning within a certain period [11]. Previous studies showed that the proportion of turnover intention among primary care providers in China ranged from 42.3 to 58.0% [12, 13]. This high turnover rate is associated with economic losses. It has been estimated that the recruitment, training, and productivity costs associated with turnover may be more than 5% of the total annual operating budget of a major medical center [14]. Furthermore, a heavier workload, reduced team morale, and lower work efficiency are unavoidable in the context of high turnover intention, and subsequently hinder work performance [15]. Turnover intention is affected by various factors, such as job satisfaction, work pressure, and burnout [12]. In addition, financial dissatisfaction has frequently been associated with turnover intention. A study involving Chinese primary care providers found low financial satisfaction was associated with turnover intention (odds ratio [OR] = 0.43, *P* < 0.001 [16]. However, it remains unclear whether other factors mediate the association between financial satisfaction and turnover intention.

Few studies have investigated the mechanism linking financial satisfaction with turnover intention among primary care providers. However, understanding this relationship is essential for developing effective measures to reduce turnover intention. Burnout is a prolonged response to chronic emotional and interpersonal job-related stressors and is characterized by emotional exhaustion, depersonalization, and reduced personal accomplishment [17]. A systematic review of the prevalence of burnout found that 67.0% of physicians had burnout [18]. As well as contributing to poor job performance, productivity decline, and high absence and turnover rates, burnout can exert a negative effect on a person’s colleagues [19]. Furthermore, burnout also negatively impacts the quality of sleep, job satisfaction, and coping strategies [20]. Many factors are known to contribute to burnout among primary care providers, including excessive workloads, clerical burdens, work-home conflicts, and financial dissatisfaction [21, 22]. Improving income levels can help reduce burnout. Perceived social support refers to people’s beliefs about the amount and quality of support they receive from their relationships and social contacts [23, 24]. Perceived social support can be viewed as individuals’ perceptions of available material, emotional, or spiritual support from other individuals or groups of people [25]. A previous study showed that financial satisfaction was associated with higher perceived social support [5]. Perceived social support has also been shown to be a predictor of burnout in Chinese healthcare providers [26]. In addition, perceived social support was found to be a reason for turnover intention, and also acted as a partial mediator between job satisfaction, burnout, and turnover intention [27].

To date, most research has addressed the dual relationships between financial satisfaction, turnover intention, perceived social support, and burnout. However, few studies have investigated the mechanism linking financial satisfaction with turnover intention among primary care providers. Therefore, this population-based study aimed to a) explore the associations between financial satisfaction and turnover intention among primary care providers, and b) test the extent to which the association between financial satisfaction and turnover intention may be mediated by perceived social support and burnout. Our findings will contribute to improving work efficiency and alleviating the shortage of primary care providers.

The conceptual model of mediation effect was used in this study [28]. In the mediation model, a relationship between the predictor and outcome variable is explained by mediation variable [28]. In this study, we included perceived social support and burnout as mediating variables. The mediation model hypothesized that perceived social support and burnout would affect by financial satisfaction and influence the association between financial satisfaction and turnover intention.

Guided by the conceptual models, we formulated a hypothesis: perceived social support and burnout serve as two mediators between financial satisfaction and turnover intention.

## Methods

### Study design and population

We conducted a cross-sectional study in four counties and three districts in Anhui province. Anhui province is a major province in the east of China with a large population. Anhui is divided into northern, central, and southern parts because of economic, cultural, and demographic differences. Anhui was the pilot province for the primary healthcare system in China. The primary care providers in this study included medical staff from community health service centers, township health centers, and village clinics.

A Multi-stage random cluster sampling method was used to select a district and a county from southern and central Anhui and one district and two counties from northern Anhui (northern Anhui has a larger population). A questionnaire was conducted with primary care providers, who were selected by cluster sampling method, in selected counties and districts. Each participant completed a self-administered questionnaire independently and anonymously, but research staff was available to address any questions. In each district, the questionnaires were collected by research staff immediately after they were completed by primary care providers. The exclusion criteria for our study were: a) worked for less than 1 year; b) selected the same option for all questions, and c) incomplete answers to the questionnaire. For the present analysis, we included 1214 participants with complete information for the variables analyzed.

Several training sessions were conducted for research staff before the investigation. The study was approved by the research ethics committee, and all participants voluntarily participated in the survey after providing their informed consent.

### Measures

#### Financial satisfaction

The independent variable was primary care providers’ financial satisfaction. Financial satisfaction was measured using one item that was developed and used in a previous study [29]. Participants were asked: “How satisfied are you with your financial situation?” Response options were: “very satisfied,” “satisfied,” “somewhat satisfied,” “dissatisfied,” and “very dissatisfied.”

#### Turnover intention

The dependent variable was primary care providers’ turnover intention. Turnover intention was measured with a modified version of a turnover intention assessment scale, which consisted of six items. The Chinese version of this scale was developed by Mickaeland et al. [30] Responses were on a four-point Likert ranging from 1 = “never” to 4 = “always.” The total score of the six items was computed as the score for turnover intention, which ranged from 6 to 24; a higher score indicated a stronger turnover intention. The Cronbach’s α was 0.881 in this study, indicating that the scale had good internal consistency.

#### Perceived social support

The 12-item Perceived Social Support Scale was used to measure perceived social support [31]. This instrument contains 12 items on three subscales: family (items 3, 4, 8, and 11), friends (items 6, 7, 9, and 12), and others (items 1, 2, 5, and 10). The items were rated with a seven-point Likert scale from 1 (“definitely disagree”) to 7 (“definitely agree”). The total score is an equally weighted sum of the 12 items, ranging from 12 to 84. A higher score indicates more perceived social support. The Cronbach’s α was 0.940 in this study, which indicated that the questionnaire was applicable.

#### Burnout

Burnout was measured using the Chinese version of the Maslach Burnout Inventory [32], which was revised by Li et al. and used in a previous study [33]. This adapted version consists of 15 items grouped on three dimensions, each with five items: emotional exhaustion, depersonalization, and reduced personal accomplishment. Responses were on a seven-point scale from 1 (never) to 7 (always), and total scores ranged from 15 to 105. The cut-off scores for emotional exhaustion, depersonalization, and reduced personal accomplishment were 25, 11, and 16, respectively. According to the cut-off scores, burnout was divided into four grades: no burnout, slight burnout, moderate burnout, and severe burnout. The Cronbach’s α for the scale was 0.816.

#### Covariables

To control for potential confounding variables, age (years), gender, and marital status were examined as adjustment variables.

### Statistical analysis

Descriptive statistics were used to summarize the distribution of participant characteristics, the frequency of occurrence of categorical variables (percentages), and the means and standard deviations (SD) of continuous variables.

**Fig. 1:**
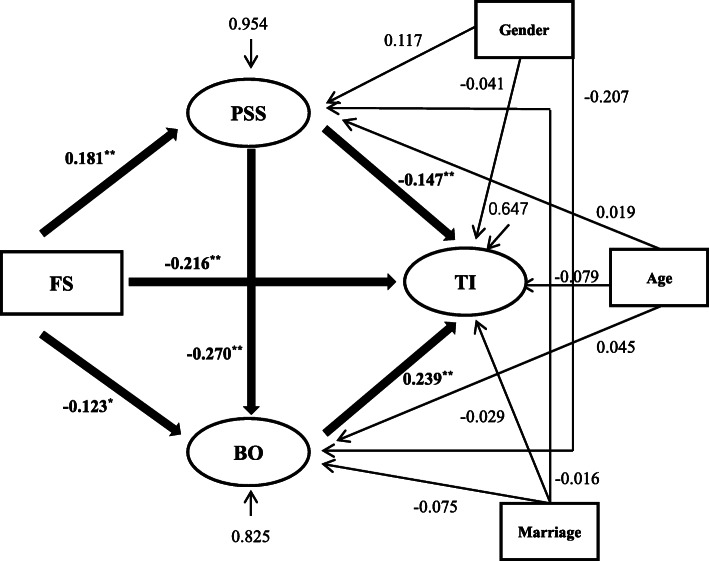
Results of the structural equation model. **p< 0.001, *p< 0.05; Adjusted for age, gender, and marital status. IS: financial satisfaction, PSS: perceived social support, BO: burnout, TI: turnover intention

Structural equation modeling (SEM) is an optimal statistical technique for evaluating a priori models, identifying mediators, and elucidating direct and indirect paths between variables. We used structural equation modeling to investigate the associations between financial satisfaction, turnover intention, and potential mediator mechanisms. Mediation analysis, which is part of structural equation analysis, was used to analyze the relationships between exposure, outcome, and mediating variables. In Fig. 1, financial satisfaction was treated as an exogenous variable, estimating two latent variables (perceived social support and burnout).

The data in this study follows a multivariate normal distribution. Maximum likelihood was used to estimate model parameters. The model fit was estimated by: a) ratio of chi-square value to degrees of freedom (χ^2^/df) ≤3; b) comparative fit index (CFI) ≥0.90; c) Tucker-Lewis index (TLI) > 0.9 d) standardized root mean square residual (SRMR) < 0.05; and e) root mean square error of approximation (RMSEA) < 0.05 [34]. Descriptive analyses were performed using SPSS 25.0 Statistics for Windows, version 25.0 (IBM Corp, Armonk, NY, USA). Mplus, version 7.4 (Muthen & Muthen, Los Angeles, CA, USA) was used for the structural equation modeling.

## Results

### Characteristics of study participants

The mean age for the 1214 participants was 40.26 (SD = 8.614) years, 55.0% were women, and 91.3% were married (6.8% were unmarried, 1.3% were divorced, and 0.7% were widowed).

We found that 6.1% of participants had no burnout, 45.0% had slight burnout, 37.6% had moderate burnout, and 11.3% had severe burnout. The mean financial satisfaction score was 2.49 (SD = 0.990) out of a possible total score of 5. The mean score for turnover intention was 14.16 (SD = 4.337), indicating that participants had a moderate turnover intention. Furthermore, the mean perceived social support score was 64.93 (SD = 13.229) (Table 1).

**Table 1:** Participants’ characteristics at baseline

Variables	Mean (SD)	n (%)
**Age**	40.26 (8.614)	
**Gender**		
Male		546 (45.0)
Female		668 (55.0)
**Marital status**		
Married		1108 (91.3)
Unmarried		82 (6.8)
Divorced		16 (1.3)
Widowed		8 (0.7)
**Financial satisfaction**	2.49 (0.990)	
**Turnover intention**	14.16 (4.337)	
**Perceived social support**	64.93 (13.229)	
**Burnout**		
No		74 (6.1)
Slight		546 (45.0)
Moderate		457 (37.6)
Severe		137 (11.3)

### Structural equation modeling

Models were built to estimate the relationships between financial satisfaction, turnover intention, perceived social support, and burnout (Fig. 1). The model fit indices and standardized direct effects among four variables and indirect effects of two mediation variables are shown in Tables 2 and 3, after adjusting gender, marital status, occupation, length of service and types of primary health care facilities. The model fit indices were evaluated as: χ^2^/df = 2.909, CFI = 0.978, TLI = 0.970, SRMR = 0.039, RMSEA = 0.040 and 90% confidence interval (CI) = 0.033 to 0.046, which showed the model had good fit.

**Table 2:** Model fit indices

Model Fit	Home
χ^2^/df	2.909
CFI	0.978
TLI	0.970
SRMR	0.039
RMSEA	0.040 (0.033, 0.046)

**Table 3:** Standardized direct and indirect effects

Variables	Standardized coefficient
**Direct effects**	
**Perceived social support**	
Financial satisfaction	0.181 (0.125, 0.234) **
Burnout	− 0.123 (−0.210, − 0.017) *
Turnover intention	− 0.216 (− 0.279, − 0.152) **
**Burnout**	
Financial satisfaction	− 0.270 (− 0.355, − 0.178) **
Turnover intention	−0.147 (− 0.217, − 0.080) **
**Turnover intention**	
Financial satisfaction	0.239 (0.140, 0.328) **
**Indirect effects**	
Financial satisfaction -- > Perceived social support -- > Turnover intention	−0.027 (− 0.042, − 0.013) **
Financial satisfaction -- > Burnout -- > Turnover intention	−0.029 (− 0.059, − 0.003) *
Financial satisfaction -- > Perceived social support -- > Burnout -- > Turnover intention	−0.012 (− 0.019, − 0.005) **
**Total Indirect**	−0.068 (− 0.100, − 0.037) **

**p < 0.001, *p < 0.05; Adjusted for age, gender, and marital status

#### Relationships among variables

In the SEM, financial satisfaction had a significant direct effect on turnover intention (β = − 0.216, 95% CI − 0.279 to − 0.152); participants with higher financial satisfaction were more likely to report low turnover intention. Financial satisfaction was significantly associated with two mediating variables. Higher financial satisfaction was associated with a decreased likelihood of having burnout (β = − 0.123, 95% CI − 0.210 to − 0.017) compared with low financial satisfaction. In terms of perceived social support, financial satisfaction often reflected higher perceived social support (β = 0.181, 95% CI 0.125–0.234) for primary care providers.

Associations were also found between turnover intention and the two mediating variables. A higher perceived social support score was associated with lower turnover intention (β = − 0.147, 95% CI − 0.217 to − 0.080). In addition, turnover intention was significantly influenced by burnout (β = 0.239, 95% CI 0.140 to 0.328), and participants with higher burnout were more likely to report higher turnout intention.

Furthermore, the association between two mediating variables (perceived social support and burnout) was significant, with low perceived social support indicating higher burnout (β = − 0.270, 95% CI − 0.355 to − 0.178). The direct and indirect effects were significant before and after the covariates were added into the model. More details are shown in Table 2.

#### Mediation role of perceived social support and Burnout

The multi-serial mediator model demonstrated that perceived social support played a potential mediating role in the association between financial satisfaction and turnover intention (β = − 0.027, 95% CI − 0.042 to − 0.013). Burnout also displayed a mediator effect in the relationship between financial satisfaction and turnover intention (β = − 0.029, 95% CI − 0.059 to − 0.003). Besides, the indirect effect on financial satisfaction and turnover intention, mediated by perceived social support to burnout, was significant (β = − 0.012, 95% CI − 0.019 to − 0.005). Overall, the three indirect pathways between financial satisfaction and turnover intention were not greater than the direct pathway. In terms of three potential mediating pathways, burnout was greater than the other two pathways.

## Discussion

This study presented critical information regarding the current profile of financial satisfaction, turnover intention, perceived social support, and burnout and their relationships among primary care providers in China. Our results showed that financial satisfaction was strongly related to turnover intention, and individuals who were dissatisfied with their financial situation had a higher risk for turnover intention However, this correlation occurred through both direct and indirect effects. Financial satisfaction had a significant indirect effect on turnover intention mediated by perceived social support and burnout. Moreover, the pathway from perceived social support to burnout played a mediating role between financial satisfaction and turnover intention.

Financial satisfaction was measured using one item in this study for the following reasons. First, one item to measure financial satisfaction has been used and its reliability has been confirmed from previous studies [27, 35–37]. In this study, to make our results more comparable with these studies, we also used one item to collect financial satisfaction data. Second, one item is more in accord with the actual situation in China. The income structure of primary care providers in China is complex, which consists of basic salary, performance salary, bonus, operational income (e.g., operate personal clinics and pharmacies), and off-the-books income. Financial satisfaction is significantly influenced by operational income and off-the-books income. However, this part of financial satisfaction is difficult to obtain due to: a) respondents refuse to answer because they do not consider this part of the income as formal earnings from work; b) respondents do not want these incomes to be known by investigators or other respondents at the same place (such as this income is illegal).

The results showed that primary care providers were generally dissatisfied with their financial situation. This finding was consistent with a previous study that found primary care providers were the least satisfied with their income [38]. Since 1980, primary care providers in China have been able to charge a 15% markup on drug sales, but this was abolished in 2017 because of over-prescribing of drugs. The government increased its subsidy for primary healthcare institutions, but this policy substantially reduced the incomes of primary care providers [2]. The mean score for turnover intention was 14.16, which was similar to previous results for general practitioners [39]. The possible interpretation for the high-level turnover intention among Chinese primary care providers is their heavy workload, low-income level, and few professional development opportunities [16]. Moreover, 11.3% of participants reported severe burnout, which was higher than that reported among Chinese primary care doctors [40] and primary care providers in the US [41]. The mean score for perceived social support in this study was 64.93, which was lower than reported in a previous study involving Chinese physicians [27].

To our knowledge, this is the first population-based study among Chinese primary care providers that investigated the relationship of financial satisfaction and turnover intention as well as the mediating roles of perceived social support and burnout. Prior studies observed correlations between financial satisfaction and turnover intention [10, 39]. However, those studies used different participant groups (e.g., specialist physicians) or analysis methods (e.g., traditional regressions and correlations). Although those studies differed from ours in terms of specific details, the results regarding the negative relationship between financial satisfaction and turnover intention were consistent, which confirms the results of our study.

This study found that financial satisfaction was associated with an increased likelihood of high perceived social support among primary care providers. A study conducted among primary healthcare patients in Brazil suggested that people with a high income were more likely to receive more perceived social support than those with a low income [42]. The workloads of primary care providers have surged since the introduction of the basic public health services program [43]. Furthermore, compared with workers with a low income, those with a high income may be able to get more support from coworkers [44]. However, income levels and workloads appear to be mismatched. Financial dissatisfaction may also reduce the desire for social intercourse and increase pressure in family life. Support from social networks may therefore be reduced by financial dissatisfaction and lack of communication. We found a correlation between low perceived social support and high risk for turnover intention, which was consistent with a previous survey of Chinese specialist physicians and emergency room nurses [27, 45]. Social relationships are established by primary care providers in daily life and also at work (e.g., physician–nurse, physician–leadership, and physician patient relationships). Primary care providers can also receive encouragement and support from their daily life networks (e.g., family and friends) or work networks (e.g., leaders and colleagues) when faced with increased workloads. These factors may explain the mediating role of perceived social support in financial satisfaction and turnover intention observed in this study. Financial dissatisfaction may lead to decreased perceived social support from family, friends, and colleagues because of lack of communication and increased life pressure, and turnover intention increases when there is a lack of perceived social support.

Financial dissatisfaction means a higher level of burnout for primary care providers; although this has not been confirmed in primary care providers, it has been described in previous studies conducted among Korean doctors [10] and Chinese nurses [46]. Moreover, an association between burnout and turnover intention was observed in this study, which was consistent with earlier findings for physicians in the United States [47]. Factors predicting burnout include low reward, excessive workload, low organizational status, and low support [48]. Medical and basic public health services provided by primary care providers that resulted in excessive workloads did not increase their income. Furthermore, there is no professional rank promotion system for primary care providers in China, which means it is difficult for them to gain a higher organizational status [49]. This may explain the associations between financial satisfaction, burnout, and turnover intention in primary care providers in this study. A mediating role of burnout between financial satisfaction and turnover intention was found in our study. Financial satisfaction was a predictor factor of burnout, and low financial satisfaction was reflected in a high level of burnout; that is, the higher level of burnout, the greater risk for turnover intention. In addition, we found a multi-serial mediation role of burnout through perceived social support between financial satisfaction and turnover intention. Financial dissatisfaction may reduce perceived social support through decreased social intercourse and increased family life pressure. Decreases in perceived social support, especially support from colleagues, may increase the risk for burnout, thereby leading to increased turnover intention.

We selected age, gender, and marital status as covariables to control potential confounding variables in the SEM. The direct and indirect effects were significant before and after the covariates were added to the model.

The strengths of this research include the use of SEM, which is an analytical method suitable for evaluation and measurement that can eliminate measurement errors for variables that are difficult to measure, such as perceived social support, burnout, and turnover intention. This study also showed a direct effect among four study variables and the mediation linking financial satisfaction with turnover intention, which is the first time this has been shown among primary care providers.

This study had several limitations. First, the measurements of financial satisfaction, turnover intention, perceived social support, and burnout were obtained using self-administered questionnaires, meaning self-report bias might have impacted the results. Second, this study was a cross-sectional study, and the interpretation of causal inferences on the results is limited. Third, the lack of work-related covariables might influence the reliability of our results. Finally, the sample was selected from one province, so the extrapolation of conclusions to the national level could be challenged. These limitations need to be addressed in further research.

## Conclusion

Our study indicates that financial dissatisfaction, low perceived social support, and burnout can aggravate turnover intention. Furthermore, perceived social support and burnout mediate the association between financial dissatisfaction and turnover intention. Our study offers an exploration of possible pathways between financial dissatisfaction and turnover intention and may have implications for reducing turnover intention among primary care providers. Ultimately, our research findings may help to alleviate the shortage of primary care providers and improve the quality of medical services. First, remuneration mechanisms need to be established to increase the income of primary care providers; for example, income could be determined by workload or professional level. Second, suitable professional promotion and organizational management systems need to be established to encourage retention among primary care providers. Finally, measures to improve perceived social support and reduce burnout are needed, such as decreased workloads and improved organizational status for primary care providers.

## Data Availability

The datasets used and/or analysed during the current study are available from the corresponding author on reasonable request.

## Abbreviation

SEM: Structural equation modeling.
